# How Health Care Professionals Evaluate a Digital Intervention to Improve Medication Adherence: Qualitative Exploratory Study

**DOI:** 10.2196/humanfactors.8948

**Published:** 2018-02-20

**Authors:** Karen Thomson, Corline Brouwers, Olga C Damman, Martine C de Bruijne, Danielle RM Timmermans, Marijke Melles

**Affiliations:** ^1^ Faculty of Industrial Design Engineering Delft University of Technology Delft Netherlands; ^2^ Department of Public and Occupational Health and Amsterdam Public Health Research Institute VU University Medical Center Amsterdam Netherlands

**Keywords:** medication adherence, eHealth, shared decision making, self-management, patient engagement

## Abstract

**Background:**

Medication nonadherence poses a serious and a hard-to-tackle problem for many chronic diseases. Electronic health (eHealth) apps that foster patient engagement and shared decision making (SDM) may be a novel approach to improve medication adherence.

**Objective:**

The aim of this study was to investigate the perspective of health care professionals regarding a newly developed digital app aimed to improve medication adherence. Familial hypercholesterolemia (FH) was chosen as a case example.

**Methods:**

A Web-based prototype of the eHealth app—MIK—was codesigned with patients and health care professionals. After user tests with patients, we performed semistructured interviews and user tests with 12 physicians from 6 different hospitals to examine how the functionalities offered by MIK could assist physicians in their consultation and how they could be integrated into daily clinical practice. Qualitative thematic analysis was used to identify themes that covered the physicians’ evaluations.

**Results:**

On the basis of the interview data, 3 themes were identified, which were (1) perceived impact on patient-physician collaboration; (2) perceived impact on the patient’s understanding and self-management regarding medication adherence; and (3) perceived impact on clinical decisions and workflow.

**Conclusions:**

The eHealth app MIK seems to have the potential to improve the consultation between the patient and the physician in terms of collaboration and patient engagement. The impact of eHealth apps based on the concept of SDM for improving medication-taking behavior and clinical outcomes is yet to be evaluated. Insights will be useful for further development of eHealth apps aimed at improving self-management by means of patient engagement and SDM.

## Introduction

### Medication Nonadherence

Medication nonadherence is a major problem faced by people with chronic conditions [1]. Nonadherence can occur both unintentionally (due to a lack of capacity or resources; eg, poor memory) and intentionally (active decision of the patient; eg, due to medication intolerance) [1,2]. The outcomes of nonadherence are well-known—loss of opportunities for patients to improve their health and the loss of medication by health care systems, with the subsequent effect of increased morbidity [3]. Identifying the principal causes of nonadherence has proven to be complex [4,5]. Medication nonadherence neither seems to be directly related to the type or severity of a disease [6] nor to individual traits or sociodemographic characteristics [7,8]. Patients’ knowledge, beliefs, and concerns regarding treatment, as well as their actual experiences with side effects do seem to be essential factors influencing medication adherence, especially *intentional* nonadherence [8-11].

### Interventions to Improve Medication Adherence

In recent years, many interventions have been developed to improve medication adherence, but these are often insufficiently successful or effective [12,13]. This is particularly seen in short-term interventions, such as counseling, written information, and personal phone calls. Long-term interventions with multiple components (eg, more convenient care, information, counseling, reminders, self-monitoring, reinforcement, family therapy, psychological therapy, mailed communications) are, in general, more likely to show benefits. However, these interventions often show a disproportional distribution between the benefits on one hand and the high expenditure of time by health care professionals and (consequent) financial resources on the other hand. Hence, there is a growing interest in digital interventions that could be time-saving [1,14]. Most digital interventions (apps), currently available for medication adherence, have functionalities such as medication reminders, medication diaries, and access to medication instructions. These apps are mainly focused on nonintentional adherence [15], and they are usually targeted only to the individual patient rather than the interaction between patients and their health care professionals. As a consequence, the health care professional’s opportunity to support patients in improving adherence may not be optimally utilized.

**Figure 1:**
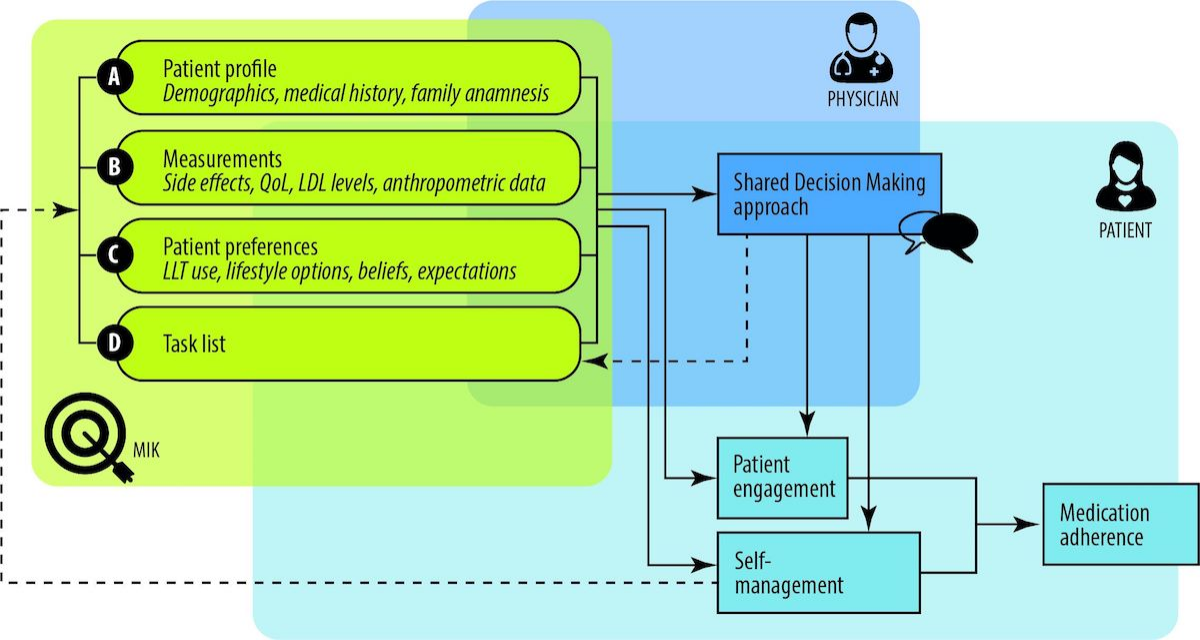
Schematic overview of functions of MIK. LLT: lipid lowering therapy; QoL: quality of life; LDL: low density lipoprotein.

To target intentional nonadherence, we developed a digital intervention in collaboration with patients and health care professionals, which focused on the patient’s preferences and beliefs about treatment options and on their actual experienced side effects and quality of life. The intervention was designed to foster patient engagement (thus, medication adherence) using 2 routes: (1) prompting patients and professionals to be aware of and discuss the patient’s preferences and beliefs about his or her current health and treatment regimen in the consultation, which is based on the Necessity–Concerns Framework (NCF) and the models of shared decision making (SDM) [8,16] and (2) increasing the patients’ engagement with management of their disease outside the consultation, through enhancing their knowledge and insight into their health status over time in relation to the medication/treatment regimen (a self-management approach) (Figure 1) [17]. By explicitly discussing patients’ beliefs, preferences, and concerns in the consultation, it seems more likely that physicians and patients choose a treatment regimen that is adhered to by the patient. Moreover, such engagement is also likely to ensure that patients take more responsibility for their health and promptly contact their physician when they encounter problems with their medication.

### Case Study: Familial Hypercholesterolemia

The genetic condition familial hypercholesterolemia (FH) was chosen as a case example for developing the digital intervention. FH patients have increased levels of low-density lipoproteins, which makes them prone for developing cardiovascular diseases (CVDs). Current estimations suggest that 1 out of every 240 people have FH [18]. Clinical guidelines state that statin medication should be the cornerstone in the treatment of FH patients [19]. In addition to statins, a considerable number of patients also need other types of lipid-lowering medications to reach optimal treatment effects (ie, reduction in the level of low-density lipoprotein). Within this regimen of lipid-lowering medications, there are decisions to be made about the type and dosage of the medications. Apart from medication, FH patients are always advised to adopt a healthy lifestyle [19].

The overall medication adherence in FH patients ranges between 58% and 89% [20,21], indicating that a substantial number of patients are nonadherent. So far, the current literature has failed to adequately explain nonadherence among FH patients [7,21]. 

As for other conditions, it is likely that adherence problems among FH patients are caused by an interplay of factors relating to patients’ beliefs, values, and experiences with side effects [1,22], as well as by factors relating to patient-professional communication [3,9,23-25]. It could be that FH patients experience a low sense of urgency because they typically do not (yet) experience actual health complaints because of FH. Additionally, the medication regimen for FH patients is lifelong and finding the right medication for FH patients is often a trial-and-error process, making medication adherence a challenge.

The developed digital app aimed to improve medication adherence of FH patients was named “MIK” (Dutch for "to aim"). After a participative human-centered design (HCD) process, involving both patients and health professionals, the final concept of MIK was first evaluated by FH patients in a pilot test. FH patients highly valued the fact that they were being triggered to think about their preferences regarding treatment and topics that they would like to discuss with their health care professional. More importantly, patients mentioned that MIK would improve their sense of control by providing an overview of important data and provide an opportunity to change their conversation with the health care professionals. The aim of this study was to investigate the perspective of health care professionals regarding MIK. The user tests and semistructured interviews addressed: (1) whether the designed functionalities aimed at improving medication adherence fit the needs of health professionals; (2) how health professionals would use and interpret the information provided by MIK; and (3) what barriers and facilitators for the use of MIK in daily practice were identified by health professionals.

## Methods

A qualitative explorative evaluation study was conducted among health care professionals to investigate their perspective regarding the designed set of eHealth functionalities in MIK, which aimed at improving medication adherence of FH patients.

### Participants

Twelve health care professionals from 6 different Dutch hospitals participated in the study. These professionals were recruited by means of a snowball sampling. All participants actively treated people with FH. The study included 6 internists, 2 internists in training, 1 rheumatologist in training, and 2 nurse practitioners. Their clinical experience ranged from 2 months to more than 10 years. Eight participants were female, and 4 were male.

### Materials

MIK was created through an iterative HCD approach [26,27], involving FH patients and health care professionals throughout the design process to ensure that the design met the needs of both user groups. A prototype of MIK was built with Invision. The advantage of this mock-up way of prototyping was that it allowed quick evaluation before putting efforts in developing the actual software. Hence, the prototype was not fully functional, but it offered an appropriate level of interactivity to have the participants experience the envisioned functionality. The prototype was built to be compatible with a computer screen-size resolution of 1920 x 993 pixels.

**Figure 2:**
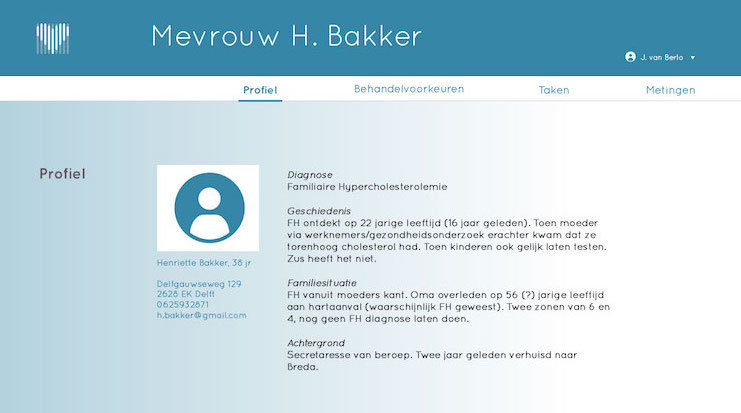
Screen patient profile.

**Figure 3:**
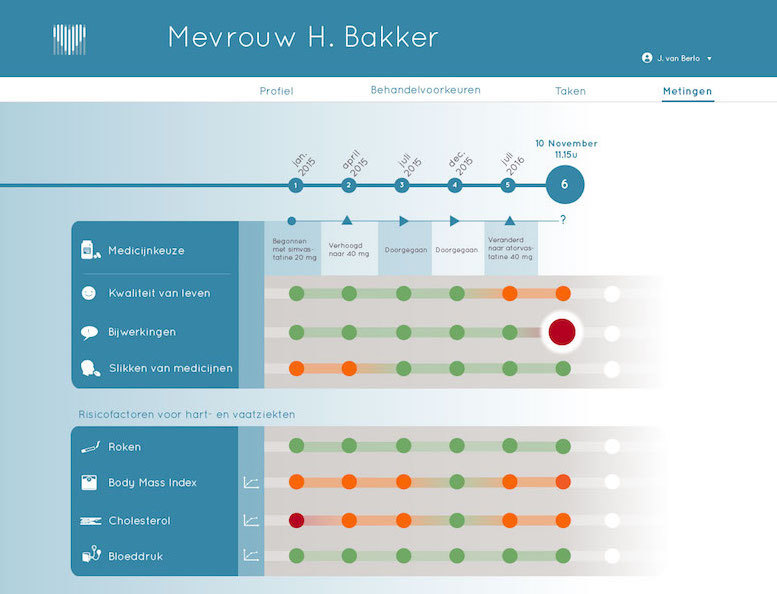
Screen measurements.

**Figure 4:**
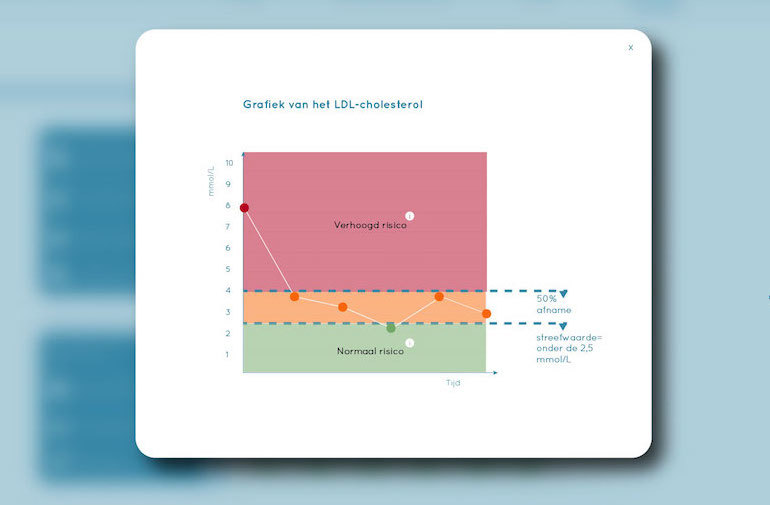
Screen cholesterol level.

**Figure 5:**
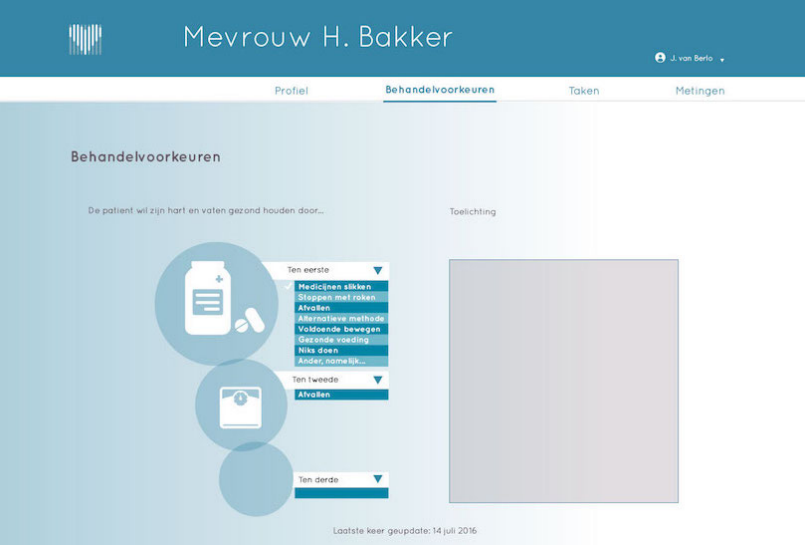
Screen treatment preferences.

**Figure 6:**
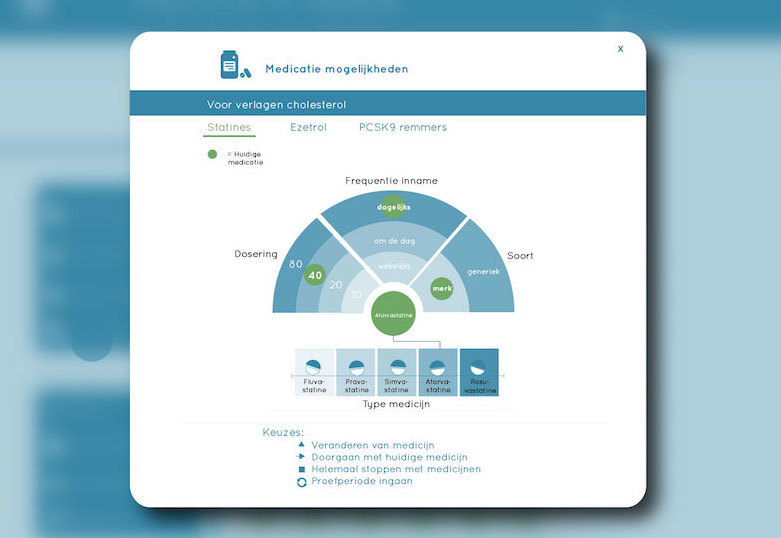
Screen medication options.

**Figure 7:**
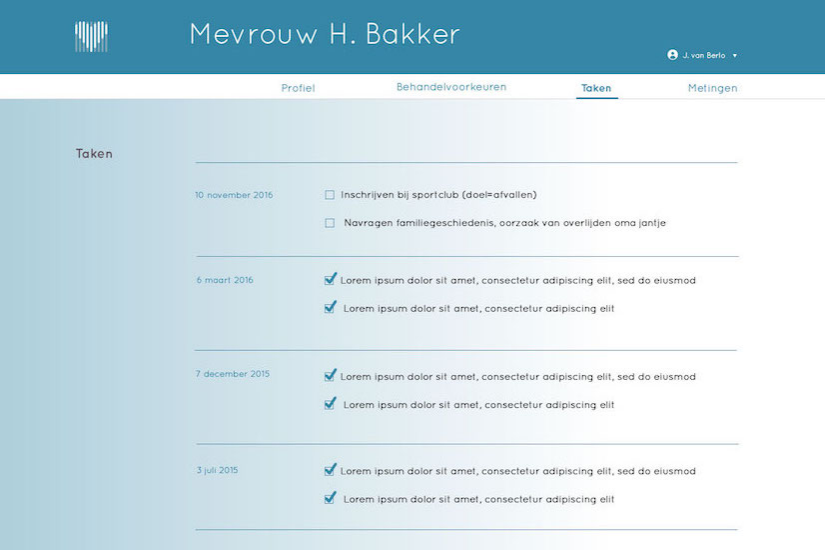
Screen task list.

**Figure 8:**
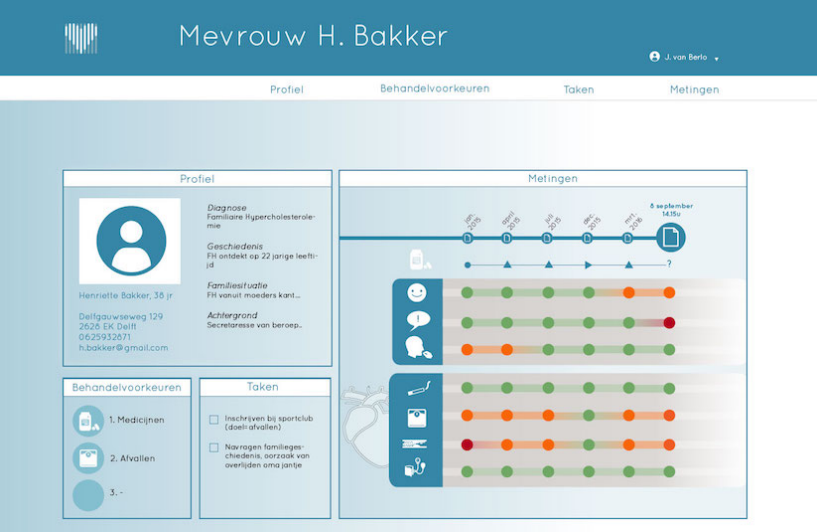
Screen patient overview.

The prototype consisted of 4 sections and an overview page:

- Patient profile, that is, details about the patient's demographics such as name, age, gender, and address, as well as basic medical information such as the diagnosis, medical history, and family anamnesis (Figure 2).
- Measurements, that is, health measurements conducted, reported, and managed by patients themselves over time. These measurements were meant to trigger patients’ necessity beliefs and concerns about side effects to be discussed in the consultation, as well as to directly foster management of their disease. This measurements section consisted of 2 main functions (Figures 3 and 4):
  - Self-reported patient information on experienced side effects, quality of life (ie, EQ-5D [28]), self-reported medication adherence, and previous medication decisions.
  - A visual overview of clinical measurements, including cholesterol levels, blood pressure (BP), and body mass index (BMI).
- Patient preferences, that is preferences of patients regarding treatment, with the aim to make patients more aware of their options and to foster a discussion about their (necessity) beliefs and concerns, and their own preferences (SDM-like approach). This section consisted of 3 main functions (Figures 5 and 6):
  - (List of) topic(s) the patient wants to discuss during the consultation: Patients are required to create a list of a top 3 topics.
  - Treatment preferences of the patient: Patients are required to create a list of the top 3 of their treatment preferences (ie, taking medication, weight loss, smoking cessation, etc); thereby prioritize the options they believe are feasible to reduce their risk of CVDs.
  - Overview of all medication options: The physician can use this feature of MIK during the consultation as a support tool to explain the dosage equivalence of different types of statins and the risk-reducing effects of medication versus lifestyle changes.
- Task list, that is a list of tasks to be agreed upon by the patient and the health care professional. This page could be used to create a list of tasks for the patient for their next consultation, decided on together with the health professional during the consultation. The list show which tasks have been completed and which ones have yet to be completed (Figure 7).
- Overview page, that is one page with the most important information at a glance. This page includes the patient profile, visual overview of the measurements, treatment preferences, and the 2 tasks that are on top of the task list (Figure 8).

### Procedure

Information about one fictional patient case was entered and presented in the prototype of MIK. This fictional case was created based on previous interviews and observations with patients in a pilot study to ensure credibility. The evaluations existed of individual sessions with health professionals, combining a user test with a semistructured interview. The user test allowed an open approach in which the participants were triggered to provide their own perspective. The researcher started with a brief introduction of the aim and context of the study. The participant was then invited to explore the overview page of the prototype. Next, the participant was provided with several task scenarios and invited to play-act these scenarios using the prototype. The researcher took the role of the patient in these playacts. The evaluation was concluded with a semistructured interview, addressing topics such as communication with and relation to the patient, information needs of the professional, implementation and integration with hospital software, and time management (Multimedia Appendix 1). Near the end of the interview, theparticipant was provided with a sheet that displayed the 8 different functions of MIK, and he or she was asked to rank their top 3 most valuable functions (Multimedia Appendix 2). A different weight was assigned to the first, second, and third most important function, as assigned by the participant, after which the sum of the weighted scores was calculated. This could help prioritize the functions that the designer could focus on and facilitate an objective discussion within the project team. The session took place at the hospital where the professional practiced. The duration of the sessions varied between 25 min and 69 min, with an average duration of 43 min.

### Analysis

Each session was audio-recorded and transcribed verbatim by the first author. A qualitative thematic analysis was performed on the data through the process of coding in 6 phases to create established, meaningful patterns: familiarization with data, generating initial codes, searching for themes among the codes, reviewing themes, defining and naming themes, and producing the final report [23]. Data were coded using the Saturate app, a Web-based tool for collaborative qualitative analysis. During the initial open coding, a total of 297 codes was generated. This large number of initial codes can be attributed to the variety of topics discussed during our semistructured interviews and the level of detail in our coding process. Consequently, axial coding was used to aggregate the codes into preliminary themes. For these preliminary themes, we used the 8 functionalities of the prototype (meaning each initial code was transferred to at least one functionality or discarded). Within each preliminary theme, we separated the codes based on whether it was a positive statement regarding the functionality or rather a statement suggesting a point of improvement regarding the functionality. Next, we decided to look for overarching themes between the functionalities that related to the design and impact of our app to provide insights that are useful for other developers in the future. This resulted in 9 subthemes relating to the perceived effect of our design (eg, making experienced complaints and side effects tangible and negotiable), the appearance of our design (eg, visualizations of clinical results over time), and information provided by our design (eg, an indication of treatment preferences). To increase the reliability of the coding process, triangulation was used. Three consensus meetings were held with 3 coauthors (CB, OD, and MM) to discuss the codes and themes. They all read 3 interviews, of which one interview was the same for each coder, to look for information in the transcripts that might be contradictory to the described themes. On the basis of these meetings, we eventually agreed upon aggregating the 9 subthemes into 3 final themes as presented below.

## Results

### Health Care Professionals’ Assessment of the Functionalities Aimed at Improving Medication Adherence

Table 1 shows the participants' assessments of the different (sub)functionalities provided by our prototype of MIK. One of the top 3 most valuable functions, as indicated by 9 out of 12 participants, was having an overview of what the patient wants to discuss during the consultation, followed by having information on side effects and quality of life as experienced by the patient (8 out of 12 participants).

### Perceived Impact of MIK on Patient-Physician Collaboration

#### Indication of Topics the Patient Prefers to Discuss

The fact that MIK offers the opportunity to the patients to highlight topics the patient wants to discuss during the consultation was considered a good starting point for the consultation by the participants. The participants argued that knowing a patient's request for help was highly important to provide optimal patient care:

This is in principle the patient's request for help at that moment in time. Therefore, I believe that is the most important part.

HCP6

#### Gaining Insight Into the Treatment Preferences of the Patient

According to the participants, insight into the patients’ preferences regarding treatment could be used to assess whether maladaptive beliefs or misconceptions exist concerning the different treatment options. If this would be the case, the health care professional could provide patient-specific information to correct these misconceptions or beliefs. In addition, the participants also thought they could help motivate the patient to achieve a certain goal when being aware of the patient preference (ie, weight loss). Knowledge concerning the patient’s treatment preferences was also considered to be of value, as there could be a discrepancy between what the patient and what the health care professional prefers regarding treatment. Participants reasoned that information would support them in forming and delivering suitable treatment advice:

Sometimes I can really be taken by surprise. I have my statins ready and the patient says, no way I am not going to take those. Then you start deliberating about how am I going to bring this across well.

HCP3

I’d like to know beforehand. We can be confronted during consultations with yes, that and this will not work and then you must improvise about what (medication) to give.

HCP11

#### Making Experienced Complaints and Side Effects Tangible and Negotiable

The feature in MIK which can collect and display information about the patients’ experienced side effects and quality of life was considered important by the participants as they know from experience that a poor quality of life or (unacceptable) side effects could interfere with adherence and, thereby, the positive effects of the treatment for FH. During the prototype test, more than half of the participants (7/12) noticed that the fictional patient presented in MIK experienced side effects, which led them to inquire about this during the consultation. According to the participants, it could prompt a more open discussion about patient’s daily functioning (Figures 2 and 8). They imagined that patients would feel less burdened and hesitant in bringing forward their complaints using the app compared with doing this face-to-face in the consultation. Moreover, addressing these issues with the patient was seen as a necessity to prevent nonadherence.

Addressing certain complaints or questions will be improved dramatically.

HCP10

Well, I believe it is a good thing that people arrive at the consultation prepared. And things are addressed in this manner. There are also people who do not give notice (about complaints). Or they do not dare. Or they are ashamed about it.

HCP10

#### Creating a Task List Together

Setting tasks together during the consultation was seen as a feature which could stimulate the patient-physician collaboration and patients’ self-management skills. A list of tasks available and visible in the consultation was considered to be a clear way to see what tasks are still pending and what tasks were already completed. It was suggested that the ability to access the task list at any place and any time could provide patients with more control while simultaneously serving as a reminder for the next consultation.

### Perceived Impact of MIK on Patient’s Understanding and Self-Management

#### Active Role for the Patient in Collecting and Providing Information

In the MIK user scenario, the patients prepared themselves at home before the consultation by filling in the information on side effects, quality of life, and treatment preferences. Participants believed this could lead to patients taking a more active role in their treatment by increasing their self-awareness regarding their condition and treatment regimen. They also argued that this would make it easier for the health care professionals in helping and encouraging patients to reach their goals (ie, losing weight).

**Table 1:** Number of participants (n=12) who qualified the different prototype functions as the first, the second, or the third most valuable in their daily practice. Between brackets the summation of the weigh factors (wf) is depicted: the most valued function receives a weighing factor of 3, the second most valued function receives a weighing factor of 2, and the third most valued function receives a weighing factor of 1.

Functionalities		Most valued function, n (wf)	Second most valued function, n (wf)	Third most valued function, n (wf)	Total, n (wf)
Overview page		2 (6)	2 (4)		4 (10)
Patient profile				1 (1)	1 (1)
**Measurements**					
	Detailed qualitative patient information (side effects, quality of life)	4 (12)	2 (4)	2 (2)	8 (18)
	Visual overview of cholesterol, BP^a^, and BMI^b^	3 (9)	1 (2)		4 (11)
**Preferences**					
	Overview of topic(s) the patient wants to discuss	3 (9)	4 (8)	2 (2)	9 (19)
	Treatment preferences		2 (4)	5 (5)	7 (9)
	Overview of all medication options			1 (1)	1 (1)
Task list			1 (2)	1 (1)	2 (3)

^a^BP: blood pressure.

^b^BMI: body mass index.

#### Visualizations of Clinical Results Over Time

Participants described the visual graphs of the changes in body weight, cholesterol, and BP over time as simple and easy in appearance and interpretation. They reasoned that the visual graphs, especially in combination with the verbal information provided during the consultation, would be useful in objectifying the results of the treatment in terms of risk-reducing effects, thereby potentially enhancing a patients’ understanding of their disease. This was considered important, as FH patients usually do not notice changes in their BP or cholesterol in day-to-day life. Interestingly, the professionals showed different ways of using the graphs during the playact consultation. The graphs were used to encourage the patient to keep up the good work (ie, complying with medication) and to make the patient more aware of the risks associated with their current weight, BP, and cholesterol levels (ie, the high risk of CVD or diabetes when keeping this weight). Participants also acknowledged the importance of including the target cholesterol level that should be achieved as per the current clinical guidelines. High, moderately high, and normal cholesterol levels were displayed using “traffic light colors,” which were considered to be an essential piece of knowledge for the patient.

Then people can review and look back, if I deliver effort, it will be rewarded in the numbers. And that can be really motivating.

HCP6

And if you take those (pills), you clearly see it is decreasing. And here you see a value in the green area. And the green area is the guideline. Because if the LDL-cholesterol is beneath the 2.5, that is really the goal of the treatment. It is the Dutch protocol and you can reach it (points at circle in green zone). If you use your medication and mind you diet.

HCP2

### Perceived Impact on Clinical Decisions and Workflow

Participants expressed ambivalent thoughts on how MIK (ie, its general usage and its different functionalities) could contribute to an effective and efficient workflow and consultation.

#### Interpreting Measurements in a Bigger Context and Over Time

Participants valued having insight into the various measurements over time (Figure 2). They argued that it could help them quickly identify connections between the measurements and other patient data (ie, quality of life score), which could optimize their clinical decision making (eg, changing the type of statins used or referring patients to a dietician or psychologist for support in losing weight or how to cope with their condition).

We are always looking for patterns and links, and there has been a change in medication and I see some colours have changed. This could mean there is a causal relation

In addition, participants felt supported by having all patient information together in one overview (the measurements overview page presented information about the patient's medication history, experienced side effects, quality of life, medication adherence, smoking, BMI, cholesterol, and BP). They argued that this could help them prioritize what topics need their attention before the start of the consultation.

I believe that, look if someone is not feeling well, it can just be difficult to stick to therapy. So in this case I would definitely ask and see how, well, what we can do about it and if we should directly act on it.

HCP9

I am here to improve someone's health, but something that bothers the patient enormously can also be in the way of the medical treatment. So that is something that you should be able to address (about quality of life).

HCP5

#### More Effective and Tailored Consultation

Participants believed that MIK could make the 15-min consultation with FH patients more effective, as they would be able to spend less time asking the patient standard questions about the patients’ well-being. With MIK, patients would have already answered those questions and, therefore, important information could be reviewed by the health professional before the consultation. Together with the overview of all measurements, participants believed this would positively contribute to an effective and efficient preparation of the consultation. Another positive aspect was that the health care professionals would be more aware of the topics the patient wants to discuss. This could be time-saving and, thus, be an incentive for the health care professional to use the app.

I think that if I know what the patient wants to discuss, if can save me time. Sometimes it can take a while before the word is out. And now it can be much more efficient, if we know immediately what we want to address. That would be a reason for me to look beforehand for 2 seconds like...are there any highlights that need to be addressed.

HCP6

#### Additional Workload

Participants described various undesirable aspects of using an app that runs separately from the electronic patient record system. A major negative aspect was having to work with 2 systems, the electronic patient record system and MIK. They did not prefer a situation in which extra actions would be required, such as logging in and finding the right patient in MIK.

Two systems, that I would find a big disadvantage. You notice this now with many apps, all need extra actions, so that would be the biggest drawback.

HCP11

Even though most of the work of the extra registration would be on behalf of the patient, some participants disliked the fact that there would be a double registration of the laboratory values, and they would have to learn to work with a new program. The use of 2 screens in a consultation was also seen as distracting by some health care professionals, and information communication technology (ICT) prerequisites (ie, results not coming through) were regarded as something to be avoided. Besides the potential extra system and the potential distraction of having an extra screen, one participant feared that patients would expect their physician to read and act upon all the information supplied by MIK, despite the limited time available. Particularly, concerning the topic of quality of life, several participants were doubtful on how to deal with this “broad” information and considered the quality of life-related issues beyond the scope of consultation with a vascular specialist.

Something you surely want to avoid is the patient to pour out their heart in those 10 minutes of the consultation. That is something I am doubtful about.

HCP8

Large pieces of textual information submitted by the patient in the various comment boxes were considered inefficient, as this would be time-consuming to read. Additionally, one participant remarked that patients may have difficulty expressing their thoughts in writing. Another participant explained that textual information in the app might be more difficult to assess in terms of importance and severity compared with a face-to-face story. Another perceived disadvantage concerned the fact that when data from MIK would be exported and saved in the hospital system, modifications would no longer possible. In this respect, the interactivity of the app would be lost, which was argued to have a negative influence on the workflow.

#### Reliability of Data Provided by Patients

There were differences in opinions regarding the reliability of the self-reported patient data in MIK. On the one hand, health care professionals argued that registration of laboratory results was less reliable when done by the patient, while on the other hand, professionals suggested that patients would be more accurate when given the opportunity to keep track of their own data.

## Discussion

### Principal Findings

This explorative qualitative study investigated the perspective of health care professionals concerning a newly developed digital app—MIK—aimed at improving the medication adherence of patients with FH. By means of the 4 functionalities in the app (ie, patient profile; health measurements [experienced side effects and quality of life]; treatment preferences; and task list), MIK was targeted at improving patient engagement, self-management, and SDM in consultation. This study showed that these targets are largely feasible, based on the perspective of professionals involved in the care for FH patients. The majority of professionals argued that the app could improve the focus and efficiency of the consultation, enhance patient engagement, and even influence the treatment decisions made, indicating a potential shift toward SDM.

Although most of the participants were positive regarding the functions of MIK, there were considerable differences between health professionals in the degree and the manner in which the information in MIK would be used in the consultation. Some professionals argued that they would use the information in MIK to engage with the patient and prompt discussions about the patients’ beliefs and concerns so as to detect or decrease intentional nonadherence. Other professionals were more likely to use the information in MIK to provide more personalized clinical treatment. While the underlying reasons behind this difference deserve further evaluation, there is not necessarily a right or wrong way of using the information in MIK. We would like to stress that SDM is a continuum rather than a fixed way of sharing decisions with patients, and it will necessarily take different forms in different decision situations [29]. In this case of FH, there is an expert agreement that taking lipid-lowering medication, especially statins, is superior to any other treatment option (ie, lifestyle changes or homeopathic products). Hence, taking statins for the treatment of FH is not considered to be a strict-preference-sensitive decision. This is different compared with situations where multiple eligible options exist from a medical perspective that involve a trade-off among different possible outcomes of each treatment [16]. However, SDM can also be an appropriate model for FH patients because the options that may be nonequivalent as per the medical experts do seem to be sensitive to the patients’ own preferences. Moreover, within the statin regimen, there are also multiple decisions to be made with respect to the type or dosage, which should obviously be discussed between patients and professionals to target intentional nonadherence. The aim of MIK is to stimulate a more open discussion about the patients’ beliefs and preferences and to take these beliefs and preferences more explicitly into account when deciding on the type and dosage of statins and lifestyle changes. Generally, the interviewed professionals indicated that they would indeed use the information in MIK to prevent or target intentional nonadherence.

Health professionals particularly embraced the information about patients’ experienced side effects and quality of life, as well as the information about patients’ treatment preferences. This information is not routinely discussed in the current care process with FH patients. According to the interviewed professionals, this information can prompt a discussion about patients’ beliefs and concerns and can correct misconceptions and fill knowledge gaps regarding the different treatment options. The positive attitude among professionals toward patient-reported outcome (PRO) measures, such as quality of life, also seems to fit the current perspectives in health care that PROs can be equally important as clinical outcomes (ie, value-based health care) [30]. Information on PROs cannot be extracted from patients’ medical records or a proxy; therefore, PROs need to be assessed in their own right [31]. The PROs may provide important additional information to professionals to reach a more individualized patient approach. Our interviewed professionals especially addressed the interconnection between patients’ quality of life and anthropometric levels (ie, cholesterol, BP, and weight) as this could indicate low (intentional) adherence.

This study showed that user testing with health professionals resulted in valuable design implications. For example, the professionals stressed the importance of (audio)–visual options for explaining different types of statin medication to increase patient’ understanding. Professionals also made specific suggestions (which were not all described in detail above), for example, about the use of specific colors and shapes to make the app more intuitive, about the presentation of a cutoff level of cholesterol in the graph and about navigation between the screens of the app. These suggestions can be used to optimize the usability of the app. However, other design implications were more focused on the integration of the app with the electronic patient record system to avoid the use of 2 screens and the need for double data entry. Unfortunately, this problem is encountered by many studies focused on eHealth innovations and not easily resolved [32,33].

### Limitations and Further Recommendations

Although our participants were practicing health professionals, we were not able to evaluate the digital app in a real consultation with patients and their professionals. Instead, we asked professionals to imagine themselves being in the hypothetical situation that they were preparing for a consultation with a fictitious patient. It is possible that in actual practice, other issues will emerge that have not been captured in our study. In addition, the fact that the tool was a Web-based mock-up rather than a fully functioning app had its limitation. While this click-through mock-up was able to show the interface and the most important features of the app, some of the option buttons were disabled. Furthermore, although the interviewed professionals worked in 6 different academic and top clinical teaching hospitals, we cannot assume their perspective is representative for all professionals treating FH patients in the Netherlands. More research is warranted to evaluate how MIK supports a larger group of professionals in practice. In addition, we strongly recommend the need to evaluate the effect of digital tools on patient outcomes such as medication adherence, satisfaction with care, health status, and morbidity. In the past few years, there has been an enormous expansion of digital tools, for example, mobile apps, to support patients in taking their medication. Recent evaluations have shown that although some of those apps are of good quality, the effectiveness of these tools with regard to patient outcomes, such as adherence, remains unknown [34]. Finally, we recommend the development of general user-centered design principles for developing eHealth apps *to optimize medication adherence. These design principles* allow research institutes and design agencies to design eHealth apps for patient engagement, self-management, and medication adherence or to enhance the applicability and usability of their eHealth tools for other health disorders.

### Conclusions

The interviewed professionals largely embraced MIK arguing that the app could improve the focus and efficiency of the consultation and even influence treatment decisions made. They particularly valued the information about patients’ experiences with side effects and about their quality of life, which is information that is not routinely discussed in the current care process but could prompt a discussion about patients’ beliefs and concerns. According to the professionals, MIK can be used to discuss the options that exist within a treatment regimen more explicitly. Professionals also acknowledged the self-management function of MIK, making connections between data would engage and motivate patients outside the consultation to adhere to their treatment.

## Appendix

Multimedia Appendix 1 Interview guide.

Multimedia Appendix 2 Function sheet.

## Abbreviations

BMI: body mass index
BP: blood pressure
CVD: cardiovascular disease
eHealth: electronic health
FH: familial hypercholesterolemia
HCD: human-centered design
ICT: information communication technology
NCF: Necessity–Concerns Framework
PROs: patient-reported outcome
SDM: shared decision making
