# Supplementary material for: How Health Care Professionals Evaluate a Digital Intervention to Improve Medication Adherence: Qualitative Exploratory Study
Source: JMIR Hum Factors. 2018 Feb 20;5(1):e7. doi: 10.2196/humanfactors.8948 (PMC5840481; doi:10.2196/humanfactors.8948)
Supplement: Multimedia Appendix 1 [file humanfactors_v5i1e7_app1.pdf]

Multimedia Appendix 1 - Interview guide

PHASE I
Explore the main page of MIK (5-10 minutes)
Please try to imagine that you are logging in to MIK. You will have a consultation with Mrs. Bakker. Mrs. Bakker is a patient with familial hypercholesterolemia and she is under your treatment for the last 4 years. Mrs. Bakker already prepared for the consultation at home. After logging in to MIK you will see the main page (MIK_healthcare provider_03.2_find patient). Please examine this screen and can tell me what you think. This information is valuable since it can help to improve the use and the concept of the application.

[In case the healthcare professional remains quiet, try to stir a reaction by asking the following questions;

- What do you see? What does this tell you?
- What is your first impression?
- What do you expect to find on this screen?
- What information do you get from this screen?
- Is something unclear?

Task scenarios (10-15 minutes)
TASK: Could you please try to find out what are the highest risk factors for this patient?
TASK: Could you please try to find out wat kind of side effects this patient is having? How do you interpret this information?
TASK: Could you please try to find out if this person has the ambition to do something about their weight? How do you interpret this information?
TASK: Are there other aspects regarding this patient information that notice you? How do you interpret this information?

Please imagine that you are preparing for the consultation with Mrs. Bakker. You are already logged into MIK. The most recent cholesterol level of Mrs. Bakker can be found in MIK.

- What information will you be looking for in MIK. What information in MIK is of most interest to you and why?
- What is your opinion regarding the screens with the cholesterol levels over time and the options regarding different types of statins? Do you think this could offer support during the consultation?
- What do you think about the way the information is presented?
- Do you think MIK offers sufficient information to prepare for the consultation with Mrs. Bakker? Do you miss any information?

I would now like to go to the actual consultation with Mrs. Bakker. Mrs. Bakker comes in, you shake hands and exchange greetings and then….

- What is the first thing you would like to discuss with Mrs. Bakker and why?
- How would you explain this to Mrs. Bakker? Could you demonstrate this to me?
- How would you explain the cholesterol results?
- How would you explain that I have a high risk of cardiovascular disease?

- Would you pay attention to the treatment preferences that Mrs. Bakker filled in? If yes, in what way?

FASE II

Semi-structured questionnaire

Topic	Questions
**General perspective on MIK**	• What are the advantages and disadvantages of the use of MIK?<br>• Which conditions are essential in order to make MIK of added value for the consultation?
**Information need of healthcare professional**	• Which information or feature of MIK do you think is most useful? Why?<br>• Could you provide a top 3 with the information and/or features that you think are most useful (Appendix B)<br>• Which information do you think is least useful/redundant?
**Implementation / Integration with the electronic patient record**	• What do you think are the most important barriers for the implementation of MIK?<br>• When introducing new interventions in healthcare there is often some resistance. How do think this will be for MIK? And how could the basis of support could be increased among the healthcare professionals?<br>• By whom do you think that the application should be developed to have the highest chance of success? (i.e. hospital, health insurance company, patient organisation)<br>• What would it take to stimulate the use of MIK (i.e. clinical effect study, cost-effectivity study)
**Time investment**	• What do you think would be the impact of MIK on the time investment of the healthcare professional?<br>• Do you think MIK can stimulate the efficiency of the consultation?
**Preparation for the consultation**	• What do you think is the degree of gain you will experience when a patient as Mrs. Bakker is better prepared for the consultation?
**Barriers in the use of MIK**	• Do you think that you have the skills to use this application?<br>• The tasks described above where about Mrs. Bakker. This case was made up (bases on real patient interviews). Could you think of a patient who recently had a consultation during which the communication was challenging? Can you describe this consultation and could you describe if you think this consultation would have gone differently when using MIK?
**Communication with the patient**	• Do you think the application could support you with the communication with the patient?
**Relation with the patient**	• To what degree to you think the application can influence the healthcare professional-patient relationship?
