# Supplementary material for: How Health Care Professionals Evaluate a Digital Intervention to Improve Medication Adherence: Qualitative Exploratory Study
Source: JMIR Hum Factors. 2018 Feb 20;5(1):e7. doi: 10.2196/humanfactors.8948 (PMC5840481; doi:10.2196/humanfactors.8948)
Supplement: Multimedia Appendix 2 [file humanfactors_v5i1e7_app2.pdf]

Multimedia Appendix 2 - function sheet

Please indicate your top 3 features that you think are most useful

1. The patient profile
2. The patients’ treatment preferences
3. To create a task list with the patient
4. To have an overview of all the measurements
5. The graphs with the cholesterol, BMI and blood pressure levels over time
6. The overview with the medication options
7. Detailed information about:
    - Quality of life
    - Side effects
    - Lifestyle factors (smoking, BMI)
8. To have a clear indication of what the patient wants to discuss during the consultation
